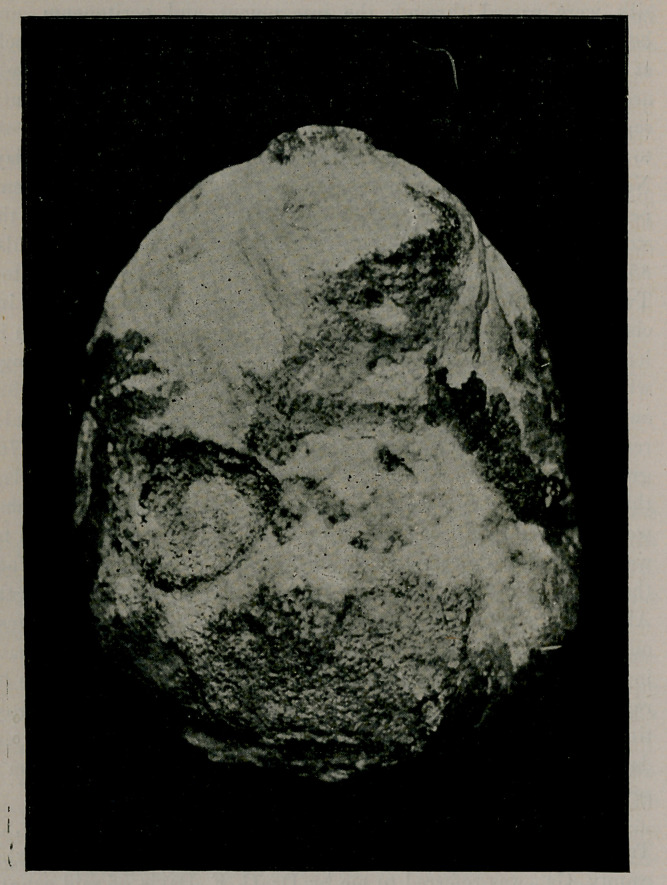# Report of Surgical Cases*Read at the meeting of the Georgia Medical Association, Savannah, April, 1895.

**Published:** 1895-08

**Authors:** W. S. Armstrong

**Affiliations:** Atlanta, Ga.; Professor of Anatomy and Clinical Surgery in Atlanta Medical College, and Surgeon to the Grady Hospital


ATLANTA

### Medical and Surgical Journal.

Vol. XII. AUGUST, 1895. No. 6.

LUTHER B. GRANDY, M.D., MANAGING EDITOR. MILLER B. HUTCHINS, M.D., BUSINESS MANAGER.

### *ORIGINAL COMMUNICATIONS.*

### REPORT OF SURGICAL CASES.*

By W. S. ARMSTRONG, M.D., Atlanta, Ga.

Professor of Anatomy and Clinical Surgery in Atlanta Medical College, and Surgeon to the Grady Hospital.

When I was requested by the chairman of the Committee on Program to prepare a paper to be read at the present session of the Medical Association of Georgia, it occurred to me that I could not present anything of more interest than several *rare* cases from my case-book, some of which I have been urged to place before the profession in the medical journals. I will, therefore, report first a case of gastrostomy.

*Gastrostomy for Impermeable Stricture of the Esophagus.*—The subject of this operation, C. W. B., was a resident of Charlotte, N. C., aged thirty, an American, and a printer by trade. He was admitted to the Grady Hospital, of Atlanta, October 27, 1892.

Some months previous to his arrival for treatment he had swallowed, through mistake, a draught of aqua ammonia, which re-

*Read at the meeting of the Georgia Medical Association, Savannah, April, 1895.

sulted in a high degree of inflammation of the mucous membrane of the mouth and esophagus. Notwithstanding the best directed efforts of treatment at his home, the accident was followed by stricture of the esophagus to such an extent that he had, for several weeks, been unable to take solid foods, and swallowed liquids with difficulty.

Upon examination with an esophageal dilator, three strictures were located, the lowest on a line with the cartilage of the fourth rib. Dilatation was practiced every second day for over three weeks, and resulted in increasing the size of the two upper, but made no impression on the lowest stricture.

During this treatment the quantity of liquids swallowed diminished daily, resulting in rapid loss of flesh and strength. Now, an operation for gastrostomy was proposed, which he declined, until a few days later, when no liquids could be swallowed.

He consented to the operation November 24, 1892. The abdomen and chest were rendered aseptic by thorough scrubbing with green soap and bichloride solution 1 to 2,000. An incision was made three and a half inches long, one inch internal to the cartilage of the eighth rib, on left side, and extending through the entire thickness of the abdominal wall. The left extremity of the liver was pressed aside, exposing the stomach, which was empty and very much contracted. A pair of forceps were introduced and the stomach drawn through the lips of the wound and held in position, while the upper and lower extremities of the wound were closed, leaving an opening one and a half inches in length. Sutures of silk were passed through the integument and peritoneum, and beneath the peritoneal and into the muscular layer of the stomach, at intervals of less than a quarter of an inch, and continued entirely around the elliptical opening. The wound was then dressed antiseptically. On the evening of the operation his temperature rose to 100½°. A nutritious enema was administered later in the day. November 25th, highest temperature 100°, pulse 80; 26th, temperature 99°, pulse 80. Ten ounces of milk introduced into the stomach by aspirator. Twenty-seventh, temperature 100°, pulse 85. The same amount of milk was again introduced by aspirator. Twenty-eighth, temperature 99¼°, pulse 81. On the 29th an incision was made through the wall of the stomach, one inch in length, and a rubber tube was introduced and secured, through which he was nourished by milk punch and soup; temperature 99° and pulse 79. Thirtieth and December 1st, temperature and pulse remained the same. December 2d, temperature normal, pulse 75; greatly improved in strength. Bowels moved naturally for the last three days, and this being the eighth day after operation the stitches were removed. He was now allowed to take solid foods, which he was required to thoroughly masticate before introducing them into the tube. From this time he gained rapidly in flesh and strength. About two months later the rubber tube was replaced by a silver one, which he has continued to use to the present time. On leaving the hospital he returned to his home in Charlotte, where he resumed his former occupation. I learn, through his letters from time to time, that he has enjoyed good health. His last letter, dated April 8, 1895, stated that he was then recovering from a severe attack of pneumonia.

*Suprapubic Operation for Vesical Calculus.*—The patient, J. J. Taylor, aged twenty-nine, a farmer, residing near Tabor, Franklin county, Ga., was referred to me by Dr. C. F. Davis of said county on December 1, 1894. His general health was poor; he was emaciated; suffered with pains in hypogastric region, and had painful and frequent urination; his temperature was elevated; pulse quick; and he stated that for three months previous, his thirst had been so intense that he drank from one to two gallons of water daily.

His mother stated that when he was an infant at the breast he gave evidence of pain accompanying urination, and from his earliest recollection he said each discharge of urine was accompanied with pain and often sudden stopping of the flow. On September 1st, last, while working on his farm, the pain in the bladder became greatly aggravated and prevented him from working up to the time of operation. Examination of the urine showed specific gravity 1,000; color turbid, reaction slightly alkaline. I examined him with Thompson's searcher, and found that he had stone in the bladder. I then directed that he should be put to bed and kept on light diet for several days.

On December 4th I performed suprapubic operation, at my surgical clinic in the presence of more than two hundred students of the Atlanta Medical College. After thoroughly cleansing the parts the bladder was washed out with a saturated solution of boracic acid; the hips were elevated, a colpeurynter was introduced into the rectum and distended with fifteen ounces of warm water. The bladder was now filled with boracic acid solution, which was retained by tying a rubber tube around the penis. An incision three and one-half inches in length was made in the linea alba down to the symphysis pubis. The hemorrhage being checked, a tenaculum was hooked into the anterior wall of the bladder and drawn forward; two strong silk ligatures were passed on either side of the hook through the walls of the bladder, and held by assistant; the bladder was opened to the full length of the external incision. Upon introducing my finger I found a calculus of unusual size attached to the posterior wall of the bladder. After detaching the stone from its position, I made an attempt to withdraw it through the incision, but found this impossible. I extended the incision upwards through the abdominal wall and bladder as high as possible without opening the peritoneal cavity, and downward through the bladder wall behind the symphysis pubis; and after several efforts removed the largest calculus I had ever seen. Now the bladder was thoroughly irrigated and the margins of the wound in the bladder wall were securely stitched to the line of incision in the integument. Then the upper and lower extremities of the wound were narrowed by deep sutures previously inserted, leaving an opening into which a large rubber tube was inserted. A light antiseptic dressing was applied and the patient removed to his room. In the afternoon his temperature reached 102½°, and pulse

120. At bedtime a dose of morphine was administered and he rested well. Each day his temperature gradually declined, and in one week was down to 98½°, with pulse from 76 to 80, and had a good appetite. The dressings were changed daily and the bladder irrigated. The wound healed rapidly and in thirty days had entirely closed, when the patient was allowed to go home. Early in March I received a letter from him stating that he had gained thirty pounds since he left Atlanta, and that he was engaged in work on his farm.

The calculus was of enormous size, elliptical in shape, flattened on two sides, and weighed at the time of removal thirteen and three-fourths ounces. Its greatest circumference was twelve inches, and its least nine and one-fourth, being larger by one-third than the one removed by Dr. J. Wm. White of Philadelphia, which has heretofore been regarded to be the largest stone successfully removed from a living person in the United States, without slough and without damaging tissues.

*Operation for Gunshot Wound in the Abdomen.*—December 13, 1894, during my service at the Grady Hospital, Richard Wallace, colored, aged eighteen, by occupation a hackman, was admitted at 3 A. M., in an inebriated condition; two hours before he was shot with a pistol, caliber unknown, but thought to be 32. The ball entered from one to two inches to the left and slightly below the umbilicus. From the direction the probe took, the ball seemed to have passed almost directly backward. The patient was conscious, pulse 100, temperature 98½°, and he was in good physical condition. Notwithstanding there was no evidence of shock, I was convinced from the course the ball had taken, that some damage had been done to the intestinal canal, and determined to make an exploratory incision, with a view to repair any injury that might be found. But before operating, I telephoned Dr. W. S. Elkin, who promptly responded, and after examining the patient, concurred with me in the propriety of making an exploratory incision, and kindly aided me in the operation. Suitable preparation of abdomen having been made, I then made an incision four to five inches long in the linea alba, below the umbilicus, when the viscera was exposed, showing a number of bloodclots, which indicated more or less hemorrhage had taken place in the cavity. Commencing with the jejunum, I carefully overlooked the intestines which were drawn out and protected by hot towels. Six perforations were found in the jejunum, and one in the mesentery. Each perforation, as soon as found, was closed with iron-dyed silk by the Lembert suture. Then the abdomen was flushed with hot sterilized water, and the wound closed and dressed.

The patient reacted nicely. He was given a tablet, of morphine, one-fourth of a grain; and atropia, one one hundred and fiftieth of a grain, at night, for one week, to prevent movement of bowels and to produce sleep. Nothing but water was allowed until the 17th, when he was given beef tea and chicken broth. December 21st, the eighth day, the stitches were removed; on the 22d, a half ounce of black matter passed from the rectum; on the 26th an enema was administered, which was repeated every other day until January 3d, when one ounce of oil ricini was given. After this the bowels moved daily. The temperature for the first ten days ranged from ninety-eight and one-half to a hundred degrees, and thereafter was normal. From this time the recovery was uninterrupted. He was able to be up on the 28th of December, and left the hospital January 8, 1895, fully restored to health.

---

### Laboratory Therapeutics.

Between laboratory experiment and clinical observation and study we believe there is a wide gulf fixed. The following from Semmola, of Rome, is an excellent expresssion of a great truth: "For thirty years I have maintained this fundamental principle, repeating it in every strain: Experimental medicine will never be in position to reproduce in the laboratory the diseases confronting us in nature; and therefore every so-called scientific construction of the evolution of this or that disease, which is based on the investigations of the laboratory, will remain a hypothetical structure built upon at least three-quarters of falsehood, hence forming a dangerous guide for the physician who would regard it as the true key to treatment. We may lull ourselves with no illusions in this respect, and I hope that rising physicians will satisfy themselves on this score, that they may not later become the murderers of their kind in the name of progress."

## Figures and Tables

**Figure f1:**
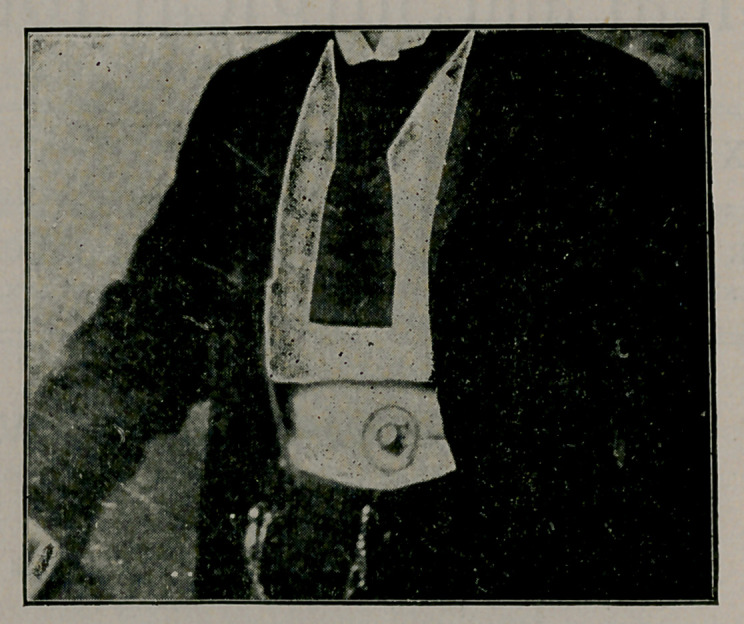


**Figure f2:**